# Supplementary material for: Health literacy among people at risk or with type 2 diabetes in Norwegian primary care—A cross sectional study
Source: PLoS One. 2024 Oct 24;19(10):e0312485. doi: 10.1371/journal.pone.0312485 (PMC11500926; doi:10.1371/journal.pone.0312485)
Supplement: Attachment — Submitted filename: response letter october 24.docx [file pone.0312485.s003.docx]

**To the editorial team,**

**PLOS ONE**

*Manuscript ID PONE-D-24-04040:" Health literacy among people at risk or with type 2 diabetes in primary care”. Thank you for reviewing our manuscript for publication in PLOS ONE, and for constructive comments. We have now revised the manuscript accordingly. Below are the comments from reviewer 1 (in bold) followed by our responses (in cursive)*.

**Reviewer 1**

**The authors have adressed most of comments.**

**In line 105 I think the term “randomization” makes a confusion about the study design. I understand that is a cross-sectional from a baseline, but, actually it is not necessary the information about the randomization here, that is not importante to this study.**

**Why did people decline the participation?**

*Thank you for this input. We agree and have removed the word “randomization” on page 105. Unfortunately we have little information on the reason for why people declined participation. We do know that the four people with manifest diabetes who withdrew their consent already were followed by the specialist health care due to a previous hospital admission.*
